# Merit of Ginseng in the Treatment of Heart Failure in Type 1-Like Diabetic Rats

**DOI:** 10.1155/2014/484161

**Published:** 2014-03-17

**Authors:** Cheng-Chia Tsai, Paul Chan, Li-Jen Chen, Chen Kuei Chang, Zhongmin Liu, Jia-Wei Lin

**Affiliations:** ^1^Department of Neurosurgery, Mackay Memorial Hospital, Taipei 104, Taiwan; ^2^Graduate Institute of Injury Prevention and Control, Taipei Medical University, Taipei 110, Taiwan; ^3^Department of Cardiology, College of Medicine, Wan Fang Hospital, Taipei Medical University, Taipei 116, Taiwan; ^4^Institute of Basic Medical Sciences, College of Medicine, National Cheng Kung University, Tainan 701, Taiwan; ^5^Department of Neurosurgery, College of Medicine, Shuang Ho Hospital, Taipei Medical University, Taipei 110, Taiwan; ^6^Department of Cardiothoracic Surgery, Shanghai East Hospital, Tongji University, Shanghai, China

## Abstract

The present study investigated the merit of ginseng in the improvement of heart failure in diabetic rats and the role of peroxisome proliferator-activated receptors **δ** (PPAR**δ**). We used streptozotocin-induced diabetic rat (STZ-rat) to screen the effects of ginseng on cardiac performance and PPAR**δ** expression. Changes of body weight, water intake, and food intake were compared in three groups of age-matched rats; the normal control (Wistar rats) received vehicle, STZ-rats received vehicle and ginseng-treated STZ-rats. We also determined cardiac performances in addition to blood glucose level in these animals. The protein levels of PPAR**δ** in hearts were identified using Western blotting analysis. In STZ-rats, cardiac performances were decreased but the food intake, water intake, and blood glucose were higher than the vehicle-treated control. After a 7-day treatment of ginseng in STZ-rats, cardiac output was markedly enhanced without changes in diabetic parameters. This treatment with ginseng also increased the PPAR**δ** expression in hearts of STZ-rats. The related signal of cardiac contractility, troponin I phosphorylation, was also raised. Ginseng-induced increasing of cardiac output was reversed by the cotreatment with PPAR**δ** antagonist GSK0660. Thus, we suggest that ginseng could improve heart failure through the increased PPAR**δ** expression in STZ-rats.

## 1. Introduction

Diabetes ranks among the main risk factors in the development of chronic heart failure (CHF) [1, 2]. Many patients with CHF and hyperglycemic symptoms have accompanying abnormalities including obesity, dyslipidemia, and hypertension that also lead to structural and functional disorders of heart in cardiac dysfunction and CHF [3–6].

Ginseng varieties have been garnering increasing interest recently for their effects on the cardiovascular system [7]. It has been demonstrated that ginseng could prevent cardiac hypertrophy and heart failure through a mechanism likely involving the prevention of calcineurin activation [8] and the latter representing a key factor for myocardial hypertrophy and remodeling [9, 10].

Peroxisome proliferator-activated receptors (PPARs) are introduced as the ligand-activated transcriptional factors to regulate the expression of genes [11]. It has been classified into three subtypes: PPAR*α*, PPAR*γ*, and PPAR*δ* to modulate the gene expressions for various bioactivities [11]. PPAR*α* is expressed in tissues with a high oxidative capacity, such as liver and heart, while PPAR*γ* is observed in limited tissues, primarily the adipose tissue [11, 12]. PPAR*δ* is known to increase lipid catabolism in both adipose and muscles [11], while PPAR*δ*-dependent cardiac function is also identified [13–15]. Deletion of cardiac PPAR*δ* is mentioned to result in decreased contraction and lowered cardiac output, showing an incidence of cardiac failure [13].

A marked decrease of PPAR*δ* expression in the hearts of streptozotocin-induced hyperglycemic rats (STZ-rats) [16] has been shown. It has also been indicated that impaired relaxation is the prominent cardiac abnormality due to the depressed troponin function in the hearts of STZ-rats [17, 18]. Thus, cardiomyopathy in STZ-rats is mainly associated with the reduced PPAR*δ* expression in hearts [16].

It has been documented that cardiac agents, such as digoxin and dobutamine, can restore the cardiac contractility in diabetic rats [19–21]. Also, cardiac agent improved cardiac contraction in STZ-rats is mainly related to the increased expression of cardiac PPAR*δ* [16]. Thus, in the present study, we employed STZ-rats to investigate the merits of ginseng in the restoration of cardiac performance in diabetic rats showing heart failure.

## 2. Material and Methods

### 2.1. Materials

GSK0660 (a specific PPAR*δ* antagonist) was purchased from Santa Cruz Biotechnology, Inc. (Santa Cruz, CA, USA). Antibodies specific to PPAR*δ*, cardiac troponin I (TnI), and phospho-troponin I (p-TnI) (Ser 23/24) were all the products of Cell Signaling Technology (Beverly, MA, USA).

### 2.2. Animals

We purchased the male Wistar rats, weighing from 250 to 280 g, from the Animal Center of National Cheng Kung University Medical College. All experiments were performed under the anesthesia with 2% isoflurane and all efforts were made to minimize suffering. The animal experiments were performed in accordance with the Guide for the Care and Use of Laboratory Animals as well as the guidelines of the Animal Welfare Act.

### 2.3. Animals and Experimental Protocol

Diabetes was induced by an intravenous injection of 60 mg/kg streptozotocin (STZ) [1]. Animals were considered to be useful as the plasma glucose concentration is up to 20 mmol/L or greater in addition to polyuria and other hyperglycemic features. The concentration of plasma glucose was measured by the glucose oxidase method (Quik-Lab, Ames, Miles, Inc., Elkhart, IN, USA). All studies were carried out 10 weeks after induction of diabetes in rats showing cardiomyopathy as described previously [2]. The STZ-rats received ginseng powder (150 mg/kg/day, orally) for 7 days. Another group of STZ-rats received same volume of vehicle; saline (0.9% sodium chloride, orally) was used for comparison, while the age-matched normal rats received same treatment with vehicle were taken as normal control. Then, they were anesthetized for cannulation in the right femoral artery with polyethylene catheters (PE-50). Mean arterial pressure (MAP) and heart rate (HR) were then measured in a polygraph (MP35, BIOPAC, Goleta, Calif) as described in our previous report [22]. Basically, all rats were kept under artificial ventilation while the cardiac output (CO) was calculated from the aortic blood flow, and the stroke volume (SV) was expressed as CO divided by HR according to our previous method [22]. After the experiment, hearts were isolated to rinse with ice-cold phosphate-buffered saline (PBS) and weighed.

### 2.4. Treatment of Antagonist

We used GSK0660 (1 mg/kg) as specific antagonist of PPAR*δ* as described previously [23]. GSK0660 from Tocris bioscience (Bristol, UK) dissolved in vehicle (Dimethyl sulfoxide, DMSO, 0.1%) was prepared to the desired dose in each assay. STZ-rats received ginseng powder (150 mg/kg/day, orally) for 7 days were injected with antagonist at one hour before the application of ginseng daily. Then, animals were anesthetized for determination of cardiac performance as described above.

### 2.5. Characterization of Hemodynamic* dP/dt*

We used hemodynamic *dP*/*dt* to measure the cardiac contractility as described in our previous report [24]. Basically, the pacing electrode of LV (IX-214; iWorx Systems, Inc., Dover, NH, USA) was placed in the anterior wall through the superior vena cava. After femoral artery and venous insertion using the Seldinger technique [25], pressure transducer was wired into the heart to monitor the RV, aortic, mean blood, and LV pressures. Pressure catheters and pacing leads were connected to the machine devise (iWorx Systems, Inc., Dover, NH, USA) to monitor the heart rate and to calculate hemodynamic signals. Body temperature was kept at 37.5°C in whole experiment.

### 2.6. Western Blotting Analysis

We used the ice-cold radioimmunoprecipitation assay (RIPA) buffer to extract the protein from tissue homogenates or cell lysates as described in our previous method [16]. The protein level was characterized using Biorad protein assay (Biorad Laboratories, Inc., Hercules, CA, USA). After separation of proteins (30 *μ*g) by SDS/polyacrylamide gel electrophoresis (10% acrylamide gel) through a Biorad Miniprotein II system, it was transferred to the expanded polyvinylidene difluoride membranes (Pierce, Rockford, IL, USA) with a Biorad Trans-Blot system. Then, the membranes were washed and blocked for 1 h at room temperature with 5% (w/v) skimmed milk powder according to our previous method [16]. The primary antibody reactions were performed following the manufacturer's instructions. The blots were incubated with goat polyclonal antibody (1 : 1000) to bind actin that served as the internal control. After removal of primary antibody, the washed blots were incubated with the appropriate peroxidase-conjugated secondary antibody for 2 h at room temperature. The blots were then developed using an ECL-Western blotting system (Amersham International, Buckinghamshire, UK). Each immunoblot, including PPAR*δ* (50 kDa), cardiac troponin I (28 kDa), or phospho-troponin I (28 kDa), was then quantified by a laser densitometer.

### 2.7. Statistical Analysis

Results were expressed as mean ± SE of each group. Statistical analysis was carried out using ANOVA analysis and Newman-Keuls post hoc analysis. Statistical significance was set as *P* < 0.05.

## 3. Results

### 3.1. Effects of Ginseng on Cardiac Abnormalities in Diabetic Rats

Streptozotocin (STZ) induced the characteristic symptoms of diabetes including hyperglycemia, hypoinsulinemia, and decreased body weight gain along with increased food and water intake when compared with age-matched normal rats (Table 1). The systolic pressure, diastolic pressure, and cardiac output in STZ-rats were markedly lower than those in normal rats (Table 1). The reduced systolic pressure and diastolic pressure in STZ-rats were recovered by ginseng after repeated treatments for 7 days (Table 1). The cardiac output in STZ-diabetic rats was also markedly enhanced by ginseng (Table 1). However, the ginseng-treated STZ-rats did not modify the blood glucose (Table 1). Also, ginseng did not influence the mean ratio of heart and body weight in STZ-rats as compared to the age-matched vehicle-treated STZ-rats (Table 1).

### 3.2. Effect of Ginseng on PPAR*δ* in the Heart of Diabetic Rats

**Figure 1:**
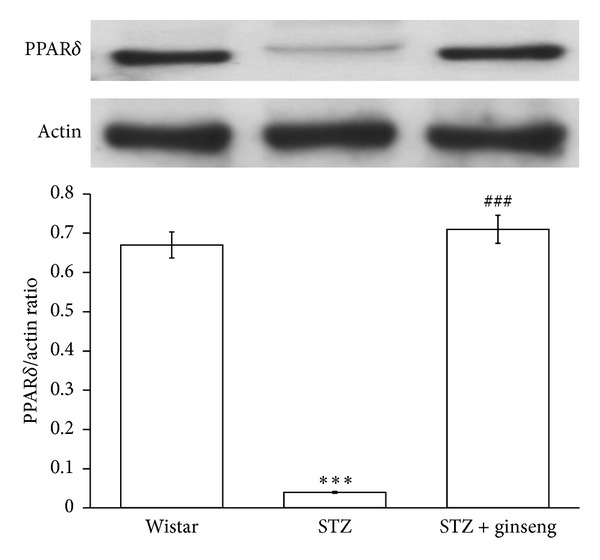
PPAR*δ* expressions in the heart isolated from vehicle-treated diabetic rats, ginseng-treated diabetic rats, or Wistar rats. Changes in PPAR*δ* expressions were investigated in age-matched Wistar rats (Control rats), vehicle-treated diabetic rats, and ginseng-treated diabetic rats. The expression of PPAR*δ* was measured using Western blotting analysis. All values are expressed as mean ± SEM (*n* = 8 per group). ***P < 0.001 as compared with Wistar rats. ^###^ *P* < 0.001 as compared with vehicle-treated diabetic rats.

The level of PPAR*δ* protein was significantly reduced in the heart of diabetic rats as compared with the normal rats (Figure 1). However, a marked increase in the expression of PPAR*δ* was observed in the heart from ginseng-treated STZ-rats (Figure 1).

### 3.3. Level of TnI Phosphorylation Was Restored by Ginseng in Diabetic Rats

**Figure 2:**
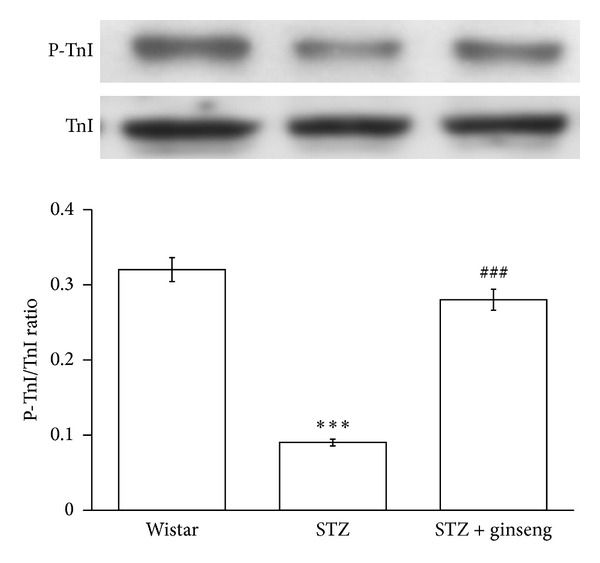
Troponin I phosphorylation in the heart isolated from vehicle-treated diabetic rats, ginseng-treated diabetic rats, or Wistar rats. Changes in Troponin I phosphorylation were investigated in age-matched Wistar rats (Control rats), vehicle-treated diabetic rats, and ginseng-treated diabetic rats. Troponin I phosphorylation was measured using Western blotting analysis. All values are expressed as mean ± SEM (*n* = 8 per group). ***P < 0.001 as compared with Wistar rats. ^###^ *P* < 0.001 as compared with vehicle-treated diabetic rats.

Change in TnI phosphorylation has been introduced to produce a profound effect on cardiac contractility and pumping [26] because phosphorylation of TnI increased cross-bridge cycling rate and enhanced the contraction power [26, 27]. The present study showed that the reduced level of TnI phosphorylation in the hearts of STZ-rats was markedly recovered by ginseng treatment (Figure 2).

### 3.4. The Recovery of Cardiac Output by Ginseng in Diabetic Rats Was Diminished by Blockade of PPAR*δ* Using GSK0660

**Figure 3:**
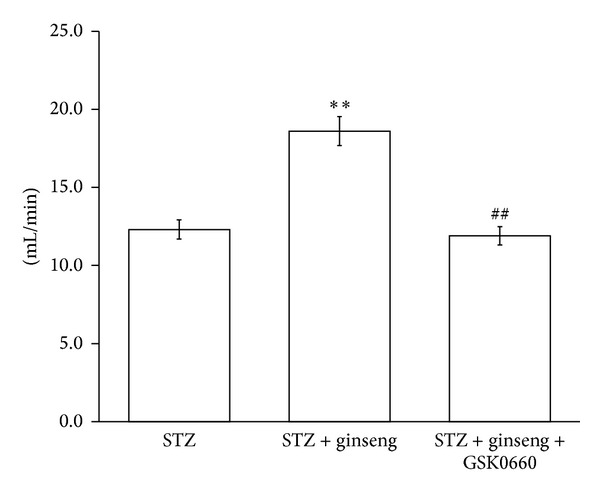
Changes of cardiac output in vehicle-treated diabetic rats (STZ), ginseng-treated diabetic rats (Ginseng-treated STZ) and ginseng-treated diabetic rats received GSK0660 (Ginseng-treated STZ + GSK0660). All values were presented as mean ± SEM (*n* = 8 per group). The ginseng-treated group was obtained from diabetic rats received the treatment of ginseng (150 mg/kg/day, orally for 7 days). **P < 0.01 as compared with vehicle-treated diabetic rats (STZ rats). The ginseng-treatment plus GSK0660 group (Ginseng-treated STZ + GSK0660) was obtained from diabetic rats received the treatment of ginseng and injected with GSK0660 (1 mg/kg) at one hour before the treatment of ginseng daily. ^##^ *P* < 0.01 as compared with the ginseng-treated diabetic rats.

Phosphorylation of cTnI is known to induce a marked increase in myofilament Ca^2+^ sensitivity and the force of cardiac contraction [28]. Thus, we investigated the cardiac output in STZ rats. The volume of cardiac output was markedly raised in ginseng treated-STZ group. But, as shown in Figure 3, this action of ginseng was inhibited by PPAR*δ* antagonist named GSK0660 at an effective dose mentioned in previous report [23].

### 3.5. The Recovery of Cardiac Contractility by Ginseng in Diabetic Rats Was Diminished by Blockade of PPAR*δ* Using GSK0660

**Figure 4:**
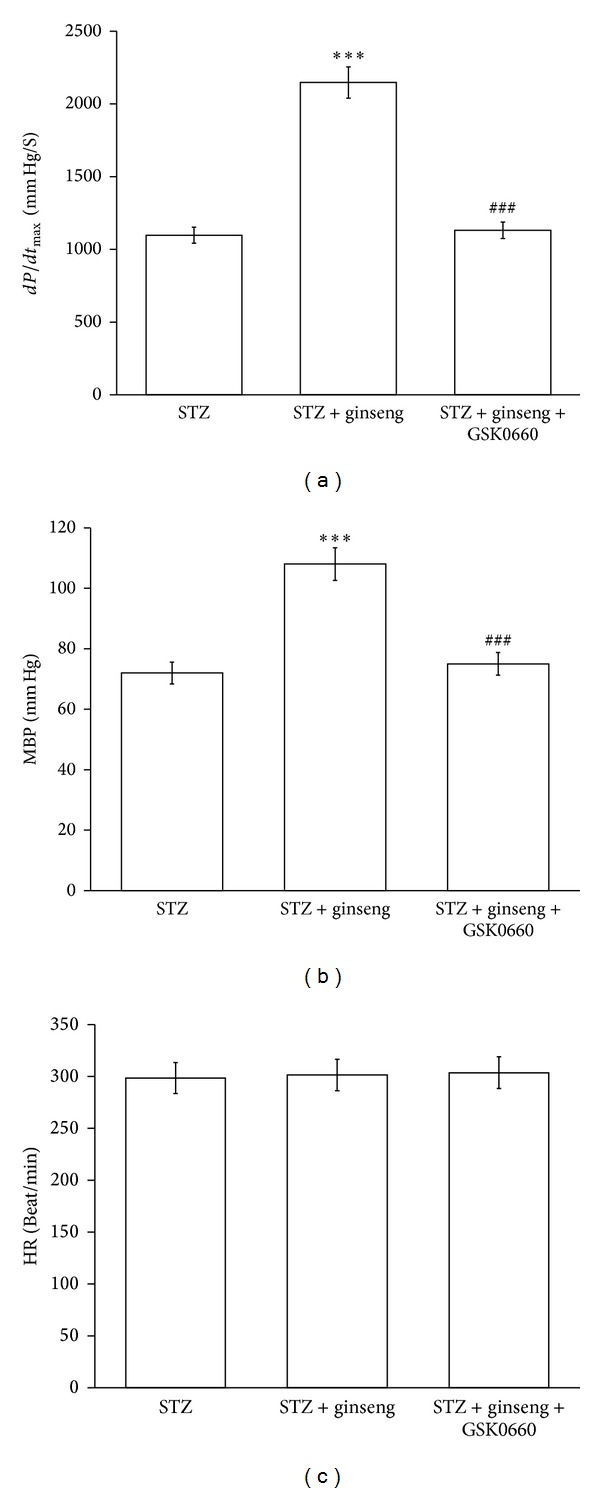
Effects of ginseng on cardiac performance in anesthetized rats. The effects of coadministration of ginseng and/or GSK0660 were investigated in the anesthetized STZ-rats. The changes in hemodynamic *dP*/*dt* (a), mean blood pressure (MBP) (b), and heart rate (HR) (c) were recorded continuously throughout the whole experiment. All values are presented as mean ± SEM (*n* = 8). ***P < 0.001 as compared to normal rats. ^###^ *P* < 0.001 as compared with the ginseng-treated diabetic rats.

The *dP*/*dt*_max⁡_ was also restored by ginseng after the repeated treatment for 7 days in STZ-rats, as compared with the vehicle-treated STZ-rats. However, as shown in Figure 4(a), this response disappeared in STZ-rats receiving coadministration of GSK0660 at the effective dose described in previous report [29]. Treatment of ginseng did not modify the heart rate but produced a slight increase in blood pressure that was also blocked by GSK0660 (Figures 4(b) and 4(c)).

## 4. Discussion

The present study found that administration of ginseng caused a marked recovery of cardiac output in addition to the lowered cardiac PPAR*δ* expression and troponin I phosphorylation in type 1-like diabetic rats. As shown in Table 1, the reduced cardiac output in diabetic rats was also markedly reversed by this repeated treatment of ginseng (150 mg/kg, orally) for 7 days that showed the most effective period. In anesthetized STZ-rats, cardiac contraction (*dP*/*dt*_max⁡_) was also significantly restored by ginseng and this change was blocked by GSK0660. However, ginseng did not modify the heart beating at this dosing. Thus, to the best of our knowledge, this is the first study to show that ginseng could restore heart failure through an activation of PPAR*δ* in type 1-like diabetic rats.

Multiple actions of ginseng are related to the treated concentrations. It has been indicated that oral administration of ginseng (12 mg/kg a daily for a 2 weeks) showed neuronal protective effect on rat with Parkinson's disease [30]. Also, rat treated with ginseng (250 or 500 mg/kg) inhibited the myocardial infarction after acute myocardial ischemia reperfusion injury [31] and isoproterenol-induced cardiac injury in rats [32]. Moreover, it was mentioned that ginseng (400 mg/kg) may enhance cardiac performance through an increase in the expression of PPAR*δ* and without altering the heart rate in normal rats [33]. In the present study, the cardiac performance in diabetic rats was also improved by repeated oral intake of ginseng at 150 mg/kg/day for one week and this used dose is markedly lower than used in previous reports for cardiac diseases [7, 8, 32, 34]. Also, this dose is equal to human oral dose about 1452 mg/kg by using the U.S. FDA HED (human equivalent dose) equation for calculation [35–37].

It has been indicated that type 1-like diabetes in STZ-induced animal is characterized by bradycardia and hypotension [38]. In conscious rats, the cardiomyopathy in this kind of animal model for heart failure was expressed by low indices of contractility and relaxation [39]. Actually, we observed the decreased cardiac *dp*/*dt* and cardiac output in STZ-induced diabetic rats similar to previous reports [22, 40].

*In vivo *and* in vitro *investigations have revealed a number of significant actions of ginsenosides and ginseng extracts in cardioprotection, such as reducing myocardial ischemia-reperfusion induced damage via NO pathway in rats and mice [41], slowing down deterioration of cardiac contractions, preventing the development of arrhythmias [42], and relaxing the muscles of the aorta [43]. Also, it has been documented that ginseng increases cardiac lipid metabolism by enhancement of PPAR*δ* expression and this action of ginseng can be blocked by the specific antagonist GSK0660 [44]. In this study, we found that ginseng could increase PPAR*δ* expression and TnI phosphorylation in the heart of diabetic rats.

It has been established that PPAR*δ* plays an important role in the regulation of cardiac performance [17–19]. In this study, we demonstrated that ginseng increases cardiac contractility without affecting heart rate in STZ-rats. Also, this cardiac tonic action of ginseng was reversed by blockade of PPAR*δ* using antagonist. Furthermore, activation of PPAR*δ* using ginseng may enhance the hemodynamic *dP*/*dt*_max⁡_ in the STZ-rats. Both actions of ginseng were inhibited by GSK0660 at a dose sufficient to block PPAR*δ* [39, 40]. The restoration of cardiac contractility in STZ-rats by ginseng through an activation of PPAR*δ* is then characterized.

The decreased level of TnI phosphorylation was reversed by ginseng in STZ-diabetic rats. Previous study showed an increase of TnI phosphorylation in rats after induction of diabetes for 8 weeks [45]. However, the reduced phosphorylation of TnI was observed in the failing heart of human studies [46]. In the present study, the reduction of TnI phosphorylation may indicate severe contractile defects in the heart of rats after induction of diabetes for 12 weeks or more. Furthermore, the lower TnI phosphorylation was also raised in the heart of STZ-diabetic rats by ginseng. Previous study indicated many phosphorylation sites on cardiac troponin I (cTnI) in physiological and pathophysiological cardiac function [47]. Studies of proteomic analysis on human heart samples taken from end-stage heart failure and rat heart samples demonstrate that Ser23/Ser24 are the major and perhaps the only sites likely to be relevant to control cardiac function [48]. Previous studies have demonstrated that TnI phosphorylation most likely acts through an enhanced off rate during Ca^2+^ exchange with TnC, leading to acceleration of relaxation and an increase in cardiac output [45, 46, 49–51]. It is suggested that the influence of ginseng on increased phosphorylation of TnI may be mediated through increasing Ca^2+^ concentrations. However, this view needs more investigations to support in the future.

The inotropic action of ginseng showed cardiac output and cardiac *dp*/*dt* was reversed by blockade of PPAR*δ* using chemical antagonist named GSK0660 as described previously [23]. In the present study, the increased cardiac output or cardiac *dp*/*dt* by ginseng was inhibited in diabetic rats receiving combined treatment with antagonist of PPAR*δ*. Thus, we conclude that activation of PPAR*δ* is involved in the ginseng-induced increase of cardiac contractility known as inotropic action. However, the effects of GSK0660 on changes of downstream signals and cardiac TnI phosphorylation or others shall be investigated in the future.

A change in heart rate is the most serious side effect of cardiac agents [41, 42]. In the present study, we showed that ginseng generated cardiac tonic action in animals without impacting the heart rate. Thus, ginseng can be used as cardiac agent without side effect of arrhythmia.

In cardiac agents, the PPAR*δ* agonist (GW0742) enhanced cardiac contractility was higher than that in the dobutamine treated samples. The increase in cardiac output caused by GW0742 was also higher than dobutamine in animals. Also, there is a slight elevation of mean blood pressure with no change of heart rate in rats treated with GW0742. This result is different to the action of dobutamine [24]. Also, the effects of ginseng on STZ rats are similar to the actions of digoxin in STZ rats and both agents restored the expression of PPAR*δ* and the cardiac contractility in STZ rats [22]. However, ginseng shows no side effect on heart rate unlike digoxin or other clinical used agents. Thus, application of ginseng to enhance cardiac performance through the activation of PPAR*δ* may be a good therapeutic strategy.

## 5. Conclusion

According to these findings, we suggest that the expression of PPAR*δ* restored by ginseng results in cardiac troponin phosphorylation in STZ-rats. Subsequently, the cardiac performance is reversed. Taken together, ginseng restored cardiac contractility through an increase in PPAR*δ* expression at the dose that did not modify the heart beating in STZ-rats. Thus, ginseng could be developed as a good cardiac agent without the side effect on heart rate in treatment of diabetic heart failure.

## Figures and Tables

**Table 1:** Characteristics of normal rats, diabetic rats, and ginseng-treated diabetic rats.

Parameters	Normal rats	Diabetic rats	Ginseng-treated diabetic rats
Food intake (g/day)	23.8 ± 3.6	42.5 ± 8.3**	43.6 ± 4.7**
Water intake (g/day)	41.5 ± 6.9	176.3 ± 11.9**	178.2 ± 11.3**
Plasma glucose (mmol/L)	5.8 ± 0.7	30.4 ± 3.8***	31.2 ± 2.8***
Body weight (g)	386.8 ± 13.5	247.6 ± 11.2**	253.4 ± 14.6**
Systolic blood pressure (mmHg)	117.3 ± 3.6	84.5 ± 8.2**	109.8 ± 4.7^##^
Diastolic blood pressure (mmHg)	81.7 ± 5.4	51.9 ± 7.2**	78.7 ± 7.6^##^
Heart rate (beats/min)	374.7 ± 23.2	303.4 ± 18.6*	297.3 ± 14.5^#^
Cardiac output (mL/min)	24.7 ± 0.5	12.4 ± 0.8*	19.2 ± 0.7^##^

Values were obtained from normal rats, vehicle-treated diabetic rats and ginseng-treated diabetic rats. All values were presented as mean ± SEM (*n* = 6 per group). The ginseng-treated group received the ginseng (150 mg/kg/day, orally for 7 days). *P < 0.05, **P < 0.01, and ***P < 0.001 as compared with normal rats. ^#^ *P* < 0.05 and ^##^ *P* < 0.01 as compared with vehicle-treated diabetic rats.
